# The Role of Maltodextrin Concentration in Maintaining Storage Stability of Dried Fruit Foams Texturized Using Plant Protein–Polysaccharide Blends

**DOI:** 10.3390/foods12081673

**Published:** 2023-04-17

**Authors:** Mine Ozcelik, Ulrich Kulozik

**Affiliations:** 1 Chair of Food and Bioprocess Engineering, TUM School of Life Sciences, Technical University of Munich, 85354 Freising, Germany; 2 Food Process Engineering, TUM School of Life Sciences, Technical University of Munich, 85354 Freising, Germany

**Keywords:** raspberry, potato protein isolate, hydrocolloid, bioactive compounds, anthocyanins, snack food

## Abstract

Hydrocolloids are widely used in food processing because of their texture-forming abilities, which help to preserve the quality of sensitive compounds, e.g., in dried fruit foams, which have recently emerged in healthier alternative snacks. Our aim was to investigate the protective role of maltodextrin in improving the storage stability of fruit foams. This study evaluated the effect of maltodextrin concentrations on the stability of the following quality parameters: anthocyanins, ascorbic acid, color, texture, and sensory perception of dried foamed raspberry pulp during storage. This study compared three concentrations (5%, 15%, and 30% *w*/*w*) of maltodextrin in mixtures, evaluating their effect on the stability of these parameters over a 12-week storage period. The foam samples were stored at 37 °C to accelerate chemical reactions under vacuum packaging conditions which excluded oxygen. The addition of 30% maltodextrin to the raspberry pulp blend resulted in the highest retentions in all compounds tested, i.e., 74% for ascorbic acid and 87% for anthocyanins. Color and texture were similarly preserved. Adding 30% maltodextrin to the mixture did not negatively influence the acceptability of sensory perception. Maltodextrin thus represents an effective protective agent for preserving nutritional and sensory qualities for a longer storage period. Hence, using MD together with potato protein was optimal for enhancing the storage stability of fruit foam, which is important for the food industry.

## 1. Introduction

Current food trends are moving towards plant-based sources, expectedly more sustainable foods which reduce the environmental impact associated with animal-based foods. In particular, the consumption of snack foods, including fruit-based products with strong health-related images, has increased. Crispy fruit snacks are produced through a combination of foaming and then drying (foam mat drying). The critical deterioration in quality that sometimes occurs during the drying process can be prevented by using microwave-supported freeze drying (MWFD) [1]. However, depending on the chemical composition, the structure of the product, and the storage conditions, quality losses can occur during storage, even in vacuum-sealed packs. However, a stable food matrix generally requires a long shelf life to preserve the nutritional and sensory qualities to the best possible extent.

To provide structural stability, biopolymers are used in foamed structures [2]. They can also preserve valuable compounds by acting as thickening agents and forming a polysaccharide–protein complex through both covalent and non-covalent interactions. These effects can be explained by the formation of protein–polysaccharide complexes, depending on their physicochemical properties and environmental factors, with impacts on electrostatic interactions, hydrophobic interactions, hydrogen bonding, and steric exclusion [3,4]. Thus, depending on the concentration of each biopolymer in the matrix, the formation of protein–polysaccharide complexes leads to encapsulated bioactives and sensitive substances. Moser et al. [5] report that protein–maltodextrin (MD) mixtures improve the protection of bioactive compounds. MD plays a particularly important role in food matrices by protecting sensitive substances, e.g., ascorbic acid, anthocyanins, and aroma-active compounds, from oxidation by acting as a barrier material [6,7,8]. Anthocyanins, a common phytochemical present in raspberries, are known as natural colorants and are responsible for raspberries’ redness [9]. The stability of anthocyanins, and, thus, of the color, is vitally important for shelf life, which is negatively affected by temperature and oxygen [10]. Hence, the ratio of MD during the food formulation process determines the stability of bioactives and the degree of protection.

MD is a long-chain sugar consisting of 2–20 α (1-4)-linked D-glucose monomers [11]. MD is characterized by its chain length, i.e., degree of polymerization (DP), and its dextrose equivalent (DE) level, which is a measure of the percentage of reducing sugars in the MD relative to the percentage of glucose (100%), where the DE value can be calculated as 100/DP. Varying DE values of MD correlate with different properties, e.g., low-DE maltodextrins (≤12) exhibit higher glass transition temperatures than high-DE maltodextrins (≥21) [12], which could have an impact on the stability of the foam structure and the access of oxygen to oxidizable substances. Therefore, the glass transition temperature (T_g_) can be increased in the presence of MD with a high molecular weight and a low DE to improve product stability [13]. Jaya and Das [13] reported that the effect of MD on the stability of powders was higher than that of other additives, such as tri-calcium phosphate and glycerol mono-stearate. Moreover, molecular characteristics, mainly the chain length of MD, increase the overall structural stability of the matrix.

Previously, we assessed the impact of the FD versus MWFD drying techniques and the impact of a porous structure, with regard to foamed versus non-foamed structures, on quality changes during storage [14]. However, we did not examine the impact of various concentrations of MD on the stability of the foamed fruit in storage, which could have an effect on the observed data. In view of the knowledge and gaps described above, we, therefore, applied MD at various concentration levels to increase shelf life due to its high glass transition temperature. We applied MD for a dual purpose: to protect labile compounds and to stabilize the foam before and during drying. We hypothesized that the higher concentration of MD would increase storage stability owing to its film-forming function, which reduces oxygen’s permeability. We also hypothesized that increasing MD concentrations would lead to greater degradation for two reasons: (1) the higher porosity obtained at higher MD concentrations causes higher oxidation due to an enlarged inner surface, because MD creates a higher oxygen exposure area, even when stored under conditions with limited oxygen availability; (2) the lower residual moisture content obtained at high MD levels leads to a higher moisture uptake during storage due to the water adsorption characteristics of MD.

Our study assessed the effect of the concentration of MD on the storage stability of raspberry foams as a model fruit. We investigated the impact of various concentrations on the retention of ascorbic acid, anthocyanins, color, texture, and sensory analysis during high-temperature (37 °C) storage over a 12-week period.

## 2. Materials and Methods

### 2.1. Fabrication of Dried Raspberry Foam

#### 2.1.1. Fresh Raspberry Foam Preparation

A frozen seedless raspberry pulp provided by Mainfrucht (Gochsheim, Germany) was first defrosted at 4 °C and then tempered to 20 °C in a water bath prior to preparation of the raspberry foam formulations. The foam formulations were used while taking into consideration the results obtained in our previous research [1,15]. Potato protein (PP) (Solanic300, Veendam, The Netherlands), as a foaming agent, and citrus pectin (P), as a gelling type foam stabilizer (degree of esterification 68–76%) (Herbstreith & Fox, Neuenbürg/Württ, Germany), were added to pulp to obtain stable fruit foam. Maltodextrin DE 6 (MD) (Nutricia, Erlangen, Germany) was used as the main protective agent for storage with a secondary role as a foam stabilizer. The PP and pectin concentrations were kept constant at 5% (*w*/*w*) and 2.5% (*w*/*w*), respectively, in all recipes during testing of MD at varying concentrations (5, 15, 30% *w*/*w*). To ensure complete dissolution, the powders and raspberry pulp were gently stirred overnight at 4 °C before whipping. Prior to foaming, mixtures were tempered at room temperature (20 ± 2 °C) for 1 h. Afterwards, foams were fabricated using a Mondomix A-05 (Mondomix Holland b. v, Almere, The Netherlands). A 600 g sample of liquid mixture for each recipe was fed and transported with the pump to the mixing head while air was injected. The mixture was aerated under controlled pressure and the foam was transported to the outlet. Each foam formulation was prepared and dried in triplicate.

#### 2.1.2. Dried Raspberry Foam Preparation

**Figure 1:**
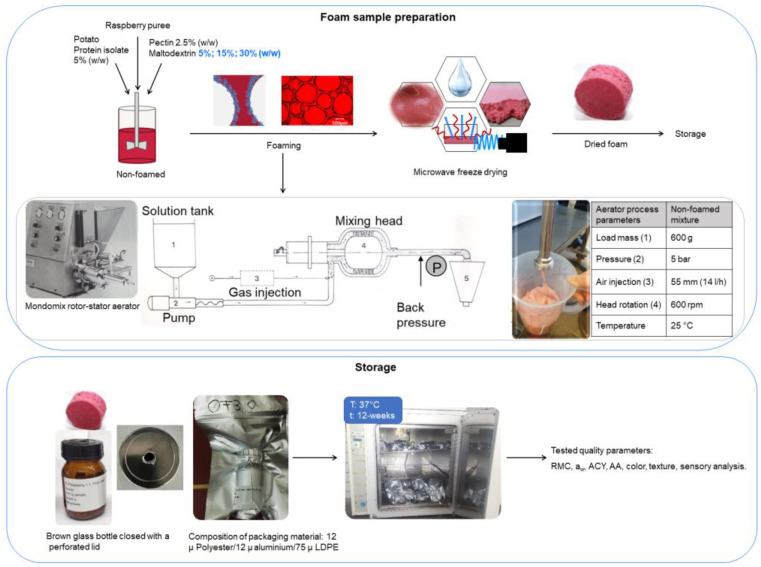
Schematic presentation of the experimental methodology with an aeration system (Haas Mondomix, Almere, The Netherlands).

A 100 g sample of fresh foam for each experiment was gently spread at a height of 1.04 ± 0.16 cm into a glass dish 190 mm in diameter (VWR International GmbH, Darmstadt, Germany) and stored at −80 °C for 24 h prior to drying. The samples were dried according to the drying procedure established in our previous research [1,16,17]. In brief, the conditions were as follows: the condenser temperature was −50 °C, the microwave power was applied using the pulsed heating mode at 1.0 W g^−1^, controlled according to the product’s surface temperature. The chamber pressure and the maximum product temperature were set to 0.1 mbar and 30 °C. A schematic diagram of the experimental method, the process diagram for the aeration system, and the storage conditions is depicted in Figure 1.

### 2.2. Storage Conditions

The dried foam samples (10 g) were placed into 250 mL cylindrical wide-necked brown glass bottles (VWR, Darmstad, Germany) and closed with perforated lids in order to maintain the foam structure. Samples were prepared in individual glass bottles for each analytical test parameter to achieve better preservation. The bottles were then vacuum-sealed (Multivac Sepp Haggenmüller SE & Co., Wolfertschwenden, Germany). Light-proof multi-layered aluminum vacuum pouches were used as packaging materials. They were made up of PET with a thickness of 12 µm, aluminum with a thickness of 12 µm, and low-density polyethylene (LDPE) with a thickness of 75 µm and a very low rate of moisture transmission **(**$\leq 0.05\;g/\left(m^{2}\cdot 24\;h\right))$ and gas permeability $\left(\leq 0.1{\;\mathrm{cm}}^{3}/\left(m^{2}\cdot 24\;h\;\cdot 0.1\;\mathrm{MPa}\right)\right)$. The samples were stored in the dark and vacuum-sealed. Each sample was packed and stored separately at 37 ± 1 °C in an incubator for 12 weeks, and was analyzed every 4 weeks starting from week 0, which was used as the control, to week 12.

### 2.3. Water Sorption Isotherms

A dynamic vapor system device (DVS) (Surface Measurement System Ltd., London, UK) was used to determine the water sorption isotherms of the samples. A 30 mg sample of the powder was used in a sample pan by applying the equilibrium method at 25 °C. The method is described in detail in Ozcelik et al. [14]. The data were fitted using a theoretical regression. The Guggenheim–Anderson–de Boer (GAB) model was applied, and calculations were made using the following equation:

(1) $$M=\frac{M_{0\;}k\;C\;a_{w}}{\left(1-k\;a_{w}\right)\left(1+\left(C-1\right)k\;a_{w}\right)}$$

where M is the equilibrium moisture content (g of water/g of solid); aw is the water activity; $M_{0}$ is the moisture content (dry basis), corresponding to an adsorbed monolayer; and C and k are the constants related to the temperature effect. For specific data regarding the C- and k-values, see the Results and Discussion sections hereinafter.

### 2.4. Determination of Color

The color parameters of the samples were measured using a SP68 Sphere Spectrophotometer color analyzer (X-Rite Europa GmbH, Regensdorf, Switzerland). In brief, the degree of lightness (L*), redness, or greenness (a*) and that of yellowness or blueness (b*) were determined, and the total color difference ΔE was calculated. The results from week 0 of each sample were used as references to evaluate the changes during the storage period. The method is described in detail in Ozcelik et al. [1].

### 2.5. Determination of Ascorbic Acid and Anthocyanin Content

Ascorbic acid was quantified by a RP-HPLC Agilent 1100 series chromatograph (Agilent Technologies, Santa Clara, CA, USA) at 243 nm. Anthocyanins, specifically cyanidin-3-sophoroside, cyanidine-3-rutinoside, and cyanidine-3-glucoside, were quantified using RP-HPLC, equipped with a Phenomenex Luna C18(2) column (Phenomenex, Aschaffenburg, Germany) to separate anthocyanins. The anthocyanin peaks were detected at 520 nm, and chromatograms were evaluated using OpenLAB CDS ChemStation software (Agilent Technologies). Both methods are described in more detail in Ozcelik et al. [1]. The quantified AA and ACY results were normalized to 100% to create a benchmark on the basis of the measurements taken following the drying of week 0 samples.

### 2.6. Texture Analysis

Foam hardness was defined as the maximum peak force (N) value obtained using a TA.TXplus texture analyzer (Stable Micro Systems, Godalming, UK) equipped with a Kramer shear cell. The five-bladed Kramer shear cell (KSC) was used for the samples subjected to a combination of compression, shearing, and extrusion; the previously established method was applied [1]. The calibration of height and weight was performed, and the distance was set to 8.5 cm prior to the measurements. A 10 g sample were used, and the crosshead speed was adjusted to 2 mm s^−1^ with a 50 kg load cell. The data were detected by Exponent 32 software (Stable Micro Systems), part of the test equipment.

### 2.7. Sensory Analysis

A quantitative descriptive analysis (QDA) was performed to investigate the influence of the MD concentration on the sensory evaluation. The sensory panelists, consisting of 10 trained assessors with experience in sensory analyses, attended several training sessions. According to Lang et al. [16], regarding the odor-active compounds in raspberry, the panelists created descriptive terms for the fruit pulp which were used for the descriptive analyses. The resulting five aroma terms are shown in Table 1. The panelists scored the intensity of each attribute using a 3-point scale (0 = not perceptible, 1 = weakly perceptible, 2 = clearly and intensively perceptible).

### 2.8. Statistical Analysis

For this study, we prepared three separate batches of each foam formulation and conducted triplicate analyses on each sample. We then calculated the mean values and standard deviations of the resulting data. We employed Origin(Pro), Version 2021 (OriginLab Corporation, Northampton, MA, USA) to conduct an analysis of variance (ANOVA) to determine the statistical differences between the tested parameters. Specifically, we conducted a one-way ANOVA (Tukey) test to assess the differences in means of the storage times and varying concentrations of maltodextrin formulations, treating these factors as independent variables. We deemed differences significant if the *p*-values were less than 0.05 (*p* < 0.05).

## 3. Results and Discussion

### 3.1. Water Sorption Behavior

The results of the residual moisture content (RMC) for the raspberry foam samples in the current study were 3.94% for 5% MD and 3.61% for 30% MD, both after 12 weeks, compared to 3.67% for 15% MD [14]. For all samples, microbiological stability was ensured, given that their water activity (a_w_) remained below 0.4 during the entire storage period.

**Figure 2:**
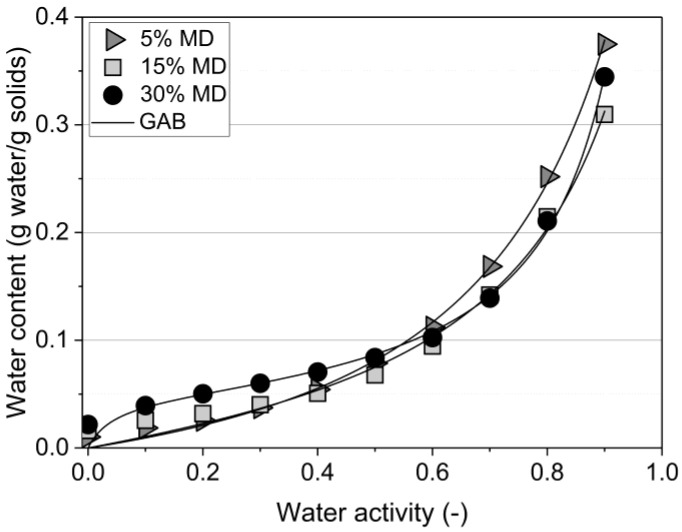
DVS (dynamic vapor sorption) curves of the tested raspberry foams at 25 °C. Foams containing 5% (*w*/*w*) PP + 2.5% (*w*/*w*) P at various MD concentrations. (GAB: —Anderson–de Boer model).

The water (ad)sorption isotherm of the samples, measured at 25 °C, is depicted in Figure 2.

The curves of samples comprising 5% and 15% MD were classified as type III according to Brunauer’s classification, thus indicating characteristics of sugar-rich fruits. Similar isotherms were observed for pineapple pulp with added MD [18]. The equilibrium moisture content was low at low water activity levels and increased sharply above a water activity level of 0.6 due to dissolving fruit sugars, in particular the high fructose content of raspberries [19,20]. In contrast, the 30% MD foam presented a sigmoidal sorption shape, type II, in which the curve was concave upwards due to the existence of multilayers on the internal surfaces. The formation of protein–polysaccharide complexes is primarily driven by electrostatic interactions for the formation of non-covalent complexes when proteins and polysaccharides carry opposite charges [3,21]. Non-electrostatic interactions between biopolymers are mainly hydrophobic interactions, and, in addition, biopolymers can form covalent bonds depending on the composition of the environment. High-molecular-weight polysaccharides, such as MD, can easily adsorb water, mainly through hydrogen bonds formed with hydroxyl and amide groups present in the foam structure. Therefore, the chemical structure, with a 30% mixing ratio of MD cross-linked with protein–pectin (PP-P), tended to swell and bind large amounts of water due to the greater availability of active sites of MD [22]. The Guggenheim–Anderson–de Boer (GAB) sorption equation, the most commonly used statistical model for foods, was applied [23,24]. Based on the mathematical analysis using the GAB model for sigmoidal type curves, the constants should range between 0.24<k ≤ 1 and 5.67 ≤ C ≤ ∞ to fulfill the GAB requirements [25]. The k values obtained were 0.83, 0.87, and 0.96, and the C values were 0.72, 1.43, and 24.0 for the foams containing 5%, 15%, and 30% MD, respectively (see Table 2).

The GAB moisture content decreased from 14% to 5% when the MD concentration increased from 5% to 30%. There was a considerable difference between the isotherms of the samples containing 5% and 15% MD at and above a_w_ = 0.6 (Figure 2). The equilibrium moisture content of the foam stabilized by 5% MD was significantly higher at the given water activity level than that at 15% MD. The presence of high concentrations of MD decreased the amount of sorbed water due to the change in the balance of hydrophilic/hydrophobic sites [26].

### 3.2. Ascorbic Acid and Anthocyanin Stability

Ascorbic acid is a highly unstable molecule and is degraded by several mechanisms, including chemical and enzymatic oxidation. After losing two electrons, AA is easily and reversibly oxidized into its DHA form. Subsequently, DHA undergoes irreversible hydrolysis to create diketogulonic acid (DKGA), leading to a complete loss of biological activity [27].

**Figure 3:**
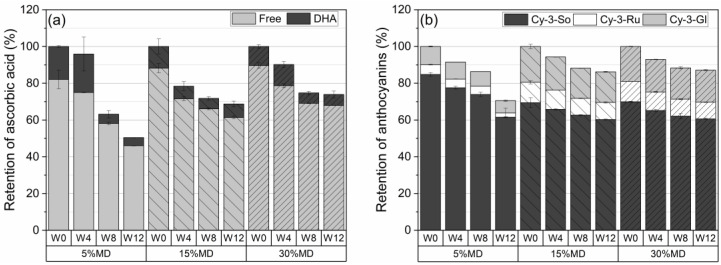
Percentage retention of (**a**) ascorbic acid and (**b**) anthocyanins in dried raspberry foams containing 5% (*w*/*w*) PP + 2.5% (*w*/*w*) P at various MD concentrations. (Free: free ascorbic acid; DHA: dehydroascorbic acid, Cy-3-So: cyanidin-3-sophoroside; Cy-3-Ru: cyanidin-3-rutinoside; Cy-3-Gl: cyanidin-3-glucoside; W: week).

Figure 3a depicts the retention percentage of AA, defined as the total of free AA and DHA at varying MD concentrations over a 12-week storage period at 37 °C. The quantified AA results were used as a benchmark to assess the decreases that occurred during storage. The increase in MD concentration resulted in a significantly (p<0.05) higher retention of AA. No significant changes were observed in the AA content of the 5% MD foam after the first 4 weeks. However, a significant decline in AA, to 37%, was measured in week 8, and this reached 50% at the end of the 12-week storage period. It was hypothesized that the low concentration of MD (5%) would only be able to provide short-term stability for labile compounds, i.e., ascorbic acid and anthocyanins, owing to the formation of a weak polysaccharide–protein complex, which would increase oxygen permeability. Thus, the thinner oxygen barrier created by the low concentration of MD at relatively high temperature conditions results in the release of AA, as well as ACY, and enhances bioaccessibility [28]. A gradual degradation of AA content was observed in the 15% MD foam, which reached 31% at 12 weeks. The highest AA retention was observed in the 30% MD foam, with a loss of 26% at 12 weeks. The 30% MD foam remained stable for AA content after week 4: the retention of AA was 74% at week 8. The film-forming function of 30% MD, which formed a denser layer, reduced oxygen permeability, hence protecting the sensitive compound. Based on the ANOVA (Tukey) test, which was performed at the 0.05 level, we found no significant differences in the population means of total ascorbic acid (AA) content among the three maltodextrin (MD) formulations during the given storage period.

Figure 3b depicts the ACY degradation of raspberry foams at varying MD concentrations. After 12 weeks, the total ACY retention values of the foams, stabilized by 5, 15, and 30% MD, were 70.6 ± 3.2%, 86.5 ± 0.4%, and 87.1 ± 0.9%, respectively. The highest retention of ACY content was obtained for the 30% MD foam due to the protective effect of MD on nutritional compounds. MD is an oligosaccharide, which is a water-soluble polysaccharide containing monosaccharides with several functions, including cell binding. An increase in MD concentration, i.e., in the space filled by the blend of raspberry pulp, in the high-molecular-weight MD rendered it nearer in vicinity to the protein, thus leading to more effective coacervation with the protein [29]. High MD concentrations (30%, *w*/*w*) formed a denser and more oxygen-impermeable wall barrier system together with the protein network, with pectin providing better stability for the sensitive substances [30]. Moreover, Moser et al. [5] reported that a higher amount of MD in grape juice powder was able to prevent the transformation of ACY to less stable forms because of the complexing of dextrins with the flavylium cation form of ACY. The fact that the 5% MD foam presented the lowest ACY and AA retention during storage also correlates with the increased redness values (a*) (see Section 3.3). According to Mishra et al. [31], the increase in the a*-values could be due to oxidation. We used ANOVA with Tukey’s test to evaluate the retention of each individual anthocyanin compound in different MD formulations during the storage period. Our results suggest that there were no significant differences among the mean values of cyanidin-3-sophoroside for the three groups (5% MD, 15% MD, and 30% MD) over the storage times, as the *p*-value for the overall ANOVA was greater than the alpha level of 0.05. However, we found a statistically significant difference in the mean values of cyanidin-3-rutinoside and cyanidin-3-glucoside across the groups over the duration of storage (*p* < 0.05).

The dual advantage of applying MD is firstly that it forms a complex with the protein, further improving foam stabilization. Second, bubbles are formed, and the foam is structurally stabilized by the protein–maltodextrin and protein–pectin networks during the vacuum-drying process. Thus, the formation of a strong network tends to protect the substances against oxidation. Once the stable foam is dried, this three-dimensional network layer acts as a protective wall, which forms on the lamella of air voids for sensitive substances.

In conclusion, the protective role of MD on the nutritional compounds, specifically AA and ACY, was exemplified in foamed matrices during high-temperature storage. Raspberry pulp mixed with a proportionate protein–carbohydrate matrix was proven to be an effective combination, especially at a higher MD concentrations, for a long-lasting dried snack with high nutritional quality.

### 3.3. Color Parameters

The color parameters of L*, a*, and b* are presented in Table 3. The redness (a*) value of 5% MD foam increased significantly (p<0.05), while the brightness (L*) value decreased. This change is likely related to the formation of anthocyanin-derived pigments, which stabilize the flavylium red-colored form [32]. Slight fluctuations in the color parameters were recorded for the foams containing 15% and 30% MD.

**Figure 4:**
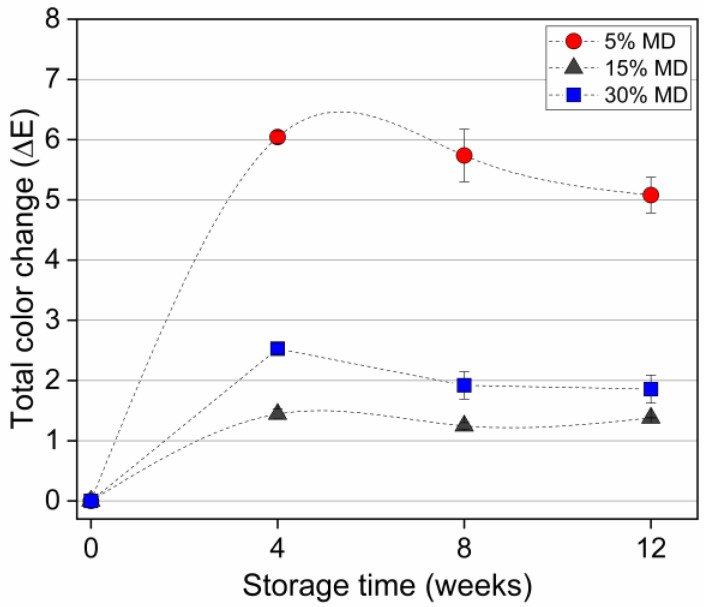
Total color changes of dried raspberry foam samples during a 12-week storage period. The foam samples contained 5% (*w*/*w*) PP + 2.5% (*w*/*w*) P at various MD concentrations (reference: week 0 of each sample).

However, we found no significant differences in color at the end of the storage period. Figure 4 depicts the total color changes in various MD foams.

A total color difference of 0 to 2 indicated that the sample was identical to the reference. Values between 2 to 5 indicated that a difference in color was detectable. The change in color (∆E*), calculated using Equation (2), was higher for the 5% MD foam, in which the difference was more evident. Increased stability of the color at higher MD concentrations indicated a positive effect of chemical stability on the pigments, corresponding with the results of a previous study on processing related color changes in red beetroot extracts [33].

### 3.4. Texture

**Figure 5:**
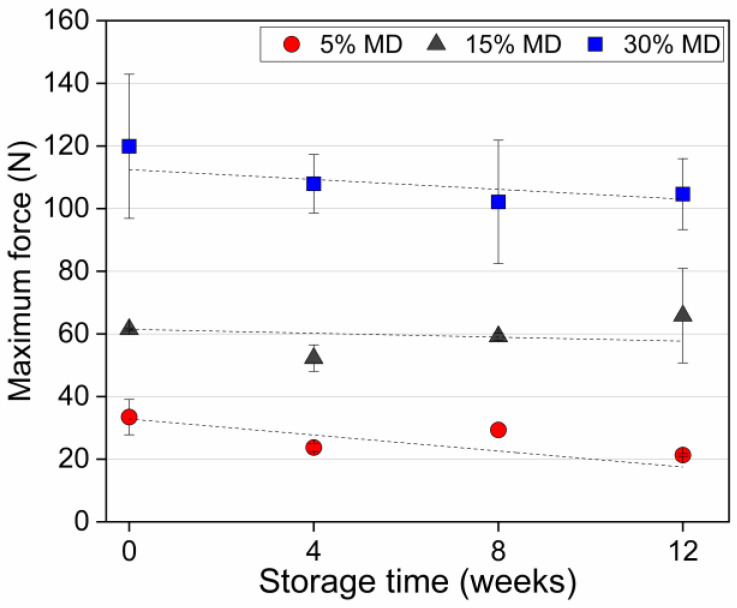
The maximum force (as a measure of sample hardness) of dried raspberry foam samples with 5% (*w*/*w*) PP + 2.5% (*w*/*w*) P at various MD concentrations.

Food texture is one of the most important drivers of taste in snack food. Structural degradation may lead to textural deformation, resulting in a crumbly and soft texture during storage. Figure 5 presents the maximum force as a measure of the hardness of dried raspberry foam samples at varying MD concentrations during storage.

The hardness value was defined as the maximum peak force of the food matrix [34]. Figure 5 clearly shows that the textural properties and stability were strongly affected by the ratio of MD relative to the other components in the samples. MD is a type of carbohydrate that can act as a thickening agent and contributes to the texture and stability of food products. When added to a foam mixture, the increased concentration of MD can lead to a denser structure and a more uniform distribution of bubbles. This is because MD molecules have multiple hydroxyl groups that can form hydrogen bonds with water molecules, creating a dense network that stabilizes the foam structure [35]. As the foam dries, the MD network becomes more concentrated, and begins to form a crystalline-like structure. This structure is highly rigid and compact, which contributes to the increased hardness of the foam. Additionally, smaller and more uniform bubble sizes and size distribution also contribute to the increased hardness, as smaller bubbles result in a higher surface area-to-volume ratio, which allows for more efficient packing of the foam structure. This efficient packing increases the density of the foam, making it harder and more stable. The smaller bubbles also reduce the size of the pores in the foam, which can limit the movement of air and water through it, further contributing to its stability and hardness. This is because the smaller pores restrict the flow of moisture and air, which can help to prevent the foam from collapsing or becoming soggy over time [1,17]. As a result, higher concentrations of MD can lead to denser foam structures with smaller bubbles, resulting in harder and more stable foam upon drying. The same trend was observed for apple gels with maltodextrin, the addition of which resulted in a firmer structure with enhanced gel strength and hardness compared to gels produced without maltodextrin [36]. A clear decreasing trend in the hardness value of the 5% MD foam was observed during the storage period. At week 0, the hardness value of the 5% MD foam was 34 N, whereas the values of 15% and 30% MD foams were 62 and 120 N, respectively. At the end of the storage period, the 5% MD foam was excessively crumbly and soft. The hardness value of the 5% MD foam significantly decreased (p<0.05) during the storage period, reaching 21 N. The moisture uptake (see Section 3.1) resulted in the development of sogginess. The decreased hardness resulted from the relatively high sugar content of the 5% MD foam (due to the low concentration of hydrocolloids), i.e., from water adsorption caused by crystalline sugar dissolution. Conversely, the hardness values of the 15% and 30% MD foams did not differ significantly (p>0.05). Based on the results of this study, it can be concluded that regulating the concentration of MD in foam-based food products can have a substantial impact on their texture and stability over the storage period. These findings hold great promise for the food industry, offering the potential to create foam-based food products with enhanced levels of quality and extended shelf-lives.

### 3.5. Sensory Analysis

**Figure 6:**
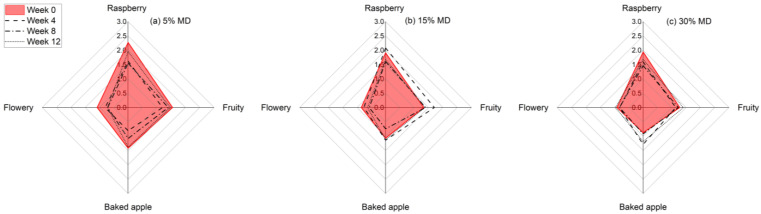
Diagram showing the average sensory ratings for the dried raspberry foam samples with 5% (*w*/*w*) PP + 2.5% (*w*/*w*) P at various MD concentrations, i.e., (**a**) 5% MD, (**b**) 15% MD, and (**c**) 30% MD, over a 12-week storage period.

In this study, we conducted a sensory evaluation of foam samples with different MD concentrations over a 12-week storage period. The sensory analysis results are shown in Figure 6. The Results indicate that the foam samples had different odor quality ratings at the beginning and end of the storage period, depending on the MD concentration.

Specifically, the foam with the lowest MD concentration had the most intense odor at the beginning, which could be attributed to the higher proportion of raspberry pulp. Our findings are consistent with previous studies that have shown that aroma intensity can be affected by storage conditions as well as the composition of the product [37]. Our results suggest that the stabilizing effect of MD on the odor of the foam samples contributed to the higher odor quality ratings observed in the samples with higher MD concentrations. This could be due to the ability of MD to bind with and stabilize aroma compounds, thus preventing their breakdown and loss of intensity over time. Additionally, the higher proportion of raspberry pulp in the foam samples with lower MD concentrations may have led to an initial spike in aroma intensity, followed by a more rapid decrease in intensity over time.

The statistical evaluation of each individual odor quality attribute for the varying MD foams demonstrated that the population means were not significantly different at the end of the storage period (*p* > 0.05).

The results of the sensory analysis suggest that the addition of MD to foam samples can have a stabilizing effect on their aroma compounds, leading to higher odor quality ratings over time. These findings have implications for the development of food products with enhanced sensory properties and longer shelf lives.

## 4. Conclusions

Nutritional and sensory qualities can be preserved by adjusting the maltodextrin ratio in the mixture. The protective role of maltodextrin in the preservation of heat- and oxygen-sensitive substances was clearly observed at higher concentrations (30%, *w*/*w*). These results indicate the encapsulation effect of maltodextrin in combination with pectin and potato protein, which, presumably, consists of the construction of an impermeable wall by forming a protein–polysaccharide network around the important compounds, thus providing longer-term stability against deterioration that is sufficient for a 12-week storage period at high temperatures. We confirmed that higher concentrations of maltodextrin increased the storage stability of the samples as a result of its film-forming and rheological function. This confirms our first hypothesis, which became evident through the increased storage stability of the dried raspberry foams in terms of their quality parameters when tested in comparison to foams with low concentrations of maltodextrin. In contrast to our second hypothesis, the higher porosity, smaller bubbles, and more uniform bubble size distribution that were obtained in the foam with 30% MD [17] did not cause significant quality degradation with regard to AA or ACY. In addition to its positive effect on nutritional preservation, adding 30% maltodextrin was determined to be acceptable in terms of sensory perception. Based on the current findings, the question that arises is whether the foam has a positive impact on the encapsulation mechanism. The changes in chemical stability and the observed color changes during the storage period indicate that an even longer shelf life, beyond 12 weeks, can be expected, as is required in commercial situations. Future research could investigate the specific mechanisms by which MD interacts with aroma compounds, and could explore the use of other stabilizers to further enhance the stability of aromas in food products.

## Figures and Tables

**Table 1:** Reference aroma substances and their concentrations used in descriptive aroma terms.

Term	Reference Compound	Composition (µg L^−1^)
Raspberry	Raspberry ketone	1846.4
Fruity	Methyl 3-methylbutanoat	194
Baked apple	β-damascenon	196
Flowery	α-ionone; β-ionone	426; 398.6

**Table 2:** Calculated GAB model parameters for the adsorption isotherms of the raspberry foams at 25 °C. The samples contained 5% (*w*/*w*) PP + 2.5% (*w*/*w*) P at various MD concentrations.

Samples	$M_{0}$ (g H_2_O/g solid d.m.)	Parameters		
		K (–)	C (–)	R^2^ (–)
5% MD	0.14 ± 0.07	0.83 ± 0.07	0.72 ± 0.43	0.99
15% MD	0.08 ± 0.03	0.87 ± 0.06	1.43 ± 0.89	0.99
30% MD	0.05 ± 0.00	0.96 ± 0.01	24.0 ± 20.10	0.99

**Table 3:** Hunter color parameters for raspberry foam samples with 5% (*w*/*w*) PP + 2.5% (*w*/*w*) P at various MD concentrations.

Storage Time Weeks	L*			a*			b*		
	5% MD	15% MD	30% MD	5% MD	15% MD	30% MD	5% MD	15% MD	30% MD
Week 0	$59.6\pm 0.6_{a}^{1}$	$59.1\pm 0.1_{a}^{1}$	$62.8\pm 0.6_{a}^{2}$	$23.2\pm 0.0_{a}^{1}$	$21.4\pm 0.6_{a}^{2}$	$20.7\pm 0.2_{a}^{2}$	$4.3\pm 0.2_{a}^{1}$	$4.5\pm 0.3_{a}^{1}$	$4.5\pm 0.0_{a}^{1}$
Week 4	$56.9\pm 0.2_{b}^{1}$	$59.7\pm 0.1_{a}^{2}$	$65.4\pm 0.1_{b}^{3}$	$28.6\pm 0.0_{b}^{1}$	$22.5\pm 0.1_{a}^{2}$	$20.8\pm 0.1_{a}^{3}$	$4.0\pm 0.3_{a}^{1}$	$5.0\pm 0.1_{a}^{2}$	$4.5\pm 0.1_{a,b}^{2}$
Week 8	$57.6\pm 0.4_{b}^{1}$	$59.7\pm 0.4_{a}^{2}$	$64.5\pm 0.4_{b}^{3}$	$28.6\pm 0.3_{b}^{1}$	$22.1\pm 0.2_{a}^{2}$	$21.4\pm 0.3_{a}^{2}$	$4.4\pm 0.2_{a}^{1}$	$5.1\pm 0.3_{a}^{1}$	$5.0\pm 0.1_{b,c}^{1}$
Week 12	$57.6\pm 0.2_{b}^{1}$	$59.6\pm 0.3_{a}^{2}$	$64.6\pm 0.3_{b}^{3}$	$27.8\pm 0.2_{b}^{1}$	$22.2\pm 0.1_{a}^{2}$	$21.1\pm 0.2_{a}^{3}$	$5.3\pm 0.1_{b}^{1}$	$5.4\pm 0.1_{a}^{1}$	$5.0\pm 0.2_{c}^{1}$

Data are presented as the mean ± standard deviation; *n* = 3. In the same column, different superscript letters indicate significant differences (*p* < 0.05) between storage times. In the same row, different subscript numerals within L*, a*, and b* indicate significant differences (*p* < 0.05) between the varying maltodextrin concentrations. The L* value denotes lightness, the a* value denotes the degree of redness, and the b* value denotes the degree of yellowness of the specimen.

## Data Availability

The data are available from the corresponding author.
